# MicroRNA‐205 is associated with diabetes mellitus‐induced erectile dysfunction via down‐regulating the androgen receptor

**DOI:** 10.1111/jcmm.14212

**Published:** 2019-02-07

**Authors:** Yan Wen, Guohui Liu, Yun Zhang, Hai Li

**Affiliations:** ^1^ Department of Endocrinology China‐Japan Union Hospital of Jilin University Changchun China; ^2^ Department of Cardiology China‐Japan Union Hospital of Jilin University Changchun China; ^3^ Department of Urology China‐Japan Union Hospital of Jilin University Changchun China

**Keywords:** androgen receptor, apoptosis, erectile dysfunction, microRNA‐205, proliferation

## Abstract

As a major class of regulatory genes in majority metazoans, microRNAs (miRs) play an important role in various diseases including diabetes mellitus (DM). Lack of androgens has previously been associated with DM‐induced erectile dysfunction (DMED). In addition, the biological functioning of androgen is mediated by androgen receptor (AR). Herein, we sought to investigate whether miRs participate in AR‐associated DMED. Sprague‐Dawlay rats were employed to establish DMED models. After modelling, levels of miR‐205 and AR in their cavernous bodies were measured. The relationship between miR‐205 and AR was verified using a dual‐luciferase reporter gene assay. The underlying regulatory mechanisms of miR‐205 were investigated in concert with the treatment of mimics or inhibitors of miR‐205, or AR overexpression in the cavernous smooth muscle cells (CSMCs) isolated from rats with DMED. Meanwhile, the effects of miR‐205 and AR on cell proliferation and apoptosis were evaluated using MTT assay and flow cytometry respectively. Rats with DMED presented with increased miR‐205 and decreased AR levels in the cavernous bodies. AR was identified as a target gene of miR‐205. Down‐regulation of miR‐205 or up‐regulation of AR could increase proliferation and inhibits apoptosis of CSMCs in addition to improvements in the erectile functioning of rats with DMED. In summary, miR‐205 may contribute to the pathogenesis of DMED via down‐regulation of AR expressions.

## 1 INTRODUCTION

Erectile dysfunction (ED) is defined as an inability to achieve or maintain an erection sufficiency for satisfactory sexual performance, and this condition commonly plagues elderly men, which affects the quality of life including psychological well‐being, family life and spousal relationship.^1^, ^2^ ED is a frequent complication of diabetes mellitus (DM).^3^, ^4^ DM represents a metabolic disorder of carbohydrate metabolism characterized by underutilized and overproduced glucose, leading to hyperglycemia.^5^ DM‐induced ED (DMED) is considered to be a result of corpus cavernous smooth muscular damage and vascular‐neuropathic injury.^6^ In addition, oxidative stress‐induced vessel and nerve lesions have been reported to play an important role in the progression of DMED.^7^ Unfortunately, the exact pathogenesis of DMED remains to be largely unknown.^8^

MicroRNAs (miRs) are 21‐23 nucleotide non‐coding RNAs that are involved in post‐transcriptional and translational regulation. The critical roles of miRs have been previously indicated in various cellular processes, such as proliferation, development and apoptosis by regulating the expression of approximately 60% of human genes.^9^

Although the role of miRs in DMED remains to be unclear, growing evidence has indicated that miRs are involved in the pathogenesis of diabetes.^10^ For instance, increased miR‐503 was reported to contribute to DM‐induced impairment of endothelial functioning and reparative angiogenesis after limb ischemia.^11^ Moreover, significantly high levels of miR‐146a were expressed in patients with type 2 DM,^12^ indicating the possible involvement of miR‐146 in type 2 DM progression.

Androgen receptor (AR) belongs to a kind of nuclear transcription factors that are activated by the steroid hormone receptor family of ligands, and contains four functional regions including a DNA‐binding domain, an amino terminal regulatory domain, a carboxy‐terminal ligand‐binding domain, and a hinge region containing a nuclear localization signal.^13^ In addition, AR mediates a wide range of cellular processes, such as proliferation, differentiation and apoptosis.^14^ Interestingly, multiple studies have indicated that AR may prevent visceral fat accumulation, while low levels of AR may serve as a potential risk factor for DM in elderly men.^15^, ^16^ Similarly, AR is highly expressed in pancreatic beta‐cell cytoplasm of control mice, and is known to gradually decrease with the progression of type 1 DM. Furthermore, the decrement of AR expression in diabetic mice was closely related to beta‐cell proliferation as well as beta‐cell apoptosis inhibition.^13^, ^14^

Interestingly, miR‐205 was previously found to be associated with the poor prognoses in patients with prostate cancer (PC) partly by negatively regulating AR.^14^, ^15^, ^16^, ^17^ However, the underlying mechanism between the involvement of miR‐205 and DMED remains to be largely unknown. Therefore, the current study aims to establish DMED rat models in order to investigate the involvement of miR‐205 in the disease development, and its further association with AR regulation.

## 2 MATERIALS AND METHODS

### 2.1 Establishment of DMED rat models

A total of 120 Sprague‐Dawley male rats (weighing 210‐240 g and aged 6‐9 weeks) were purchased from Experimental Animal Center, Sun Yat‐sen University (Guangzhou, Guangdong, China), and raised in the animal house of the China‐Japan Union Hospital of Jilin University using a 12/12 light‐dark schedule with water and food available ad libitum. The mating test showed that all rats presented with normal sexual functioning. All detailed accommodation and care procedures complied with Chinese recommendations and legislations. In addition, all efforts were made to minimize the number and suffering of the included animals. Next, the rats were randomly divided into the normal group (n = 10) and the DMED group (n = 110). After being fasted for a duration of 8‐12 hours, the rats in the DMED group were intraperitoneally injected with streptozotocin (STZ, 40 mg/kg, Sigma‐Aldrich Chemical Company, St Louis, MO, USA) in citrate buffer solution (0.1 mol/L, pH = 4.5). Simultaneously, the rats in the normal group were intraperitoneally injected with equal amounts of 0.1 mol/L citric acid‐sodium citrate buffer solution. All symptoms after the administration of STZ injection were recorded, in addition to measurement of blood glucose levels in caudal blood using the tail‐clipping method with a blood glucose meter (Roche Diagnostics, Indianapolis, IN, USA) from the 4th day. The blood glucose levels were measured once in a week. Rats presenting with random blood glucose level >16.67 mmol/L were considered to be diabetic. Protamine zinc insulin would be injected subcutaneously to the neck of rats with blood glucose >25 mmol/L, and in coma. A total of 96 rats were found to be successfully established into DM rat models. After 10 weeks of feeding, DM rats were screened, and the animals were weighed and placed into test cages. Under quiet conditions, the light in the room was diminished, so that the rats could adapt to the environment for 10 minutes. Next, a total of 100 µg/kg apomorphine (APO, Shenyang No.1 Pharmaceutical Factory, Shenyang, Liaoning, China) was subcutaneously injected into the soft skin of the neck. After administering injections, the rats were observed using a video recorder from the bottom of the cage for 30 minutes, and the erection times were recorded. The standards of penile erection in rats were as follows: the prepuce was receded, and the penis was enlarged and the glans was exposed. Abnormal erectile function was defined as no erection.^18^ Our statistical results showed that 70 DMED rats were successfully established. All aforementioned experiments were approved by the Ethics Committee of the China‐Japan Union Hospital of Jilin University.

### 2.2 Observation of erectile function in rats

One week after intervention, observations were conducted during the night in a quiet environment, and the rats were injected with 100 g/kg APO in the neck. After the rats were allowed to adapt for 5 minutes, the number of erections in rats in each group was recorded for 30 min.

### 2.3 Animal grouping and transfection

A total of 60 rats from the DMED groups were selected randomly, and divided into the following six groups (10 rats in each group): the DMED group (without any transfection), the negative control group (NC, transfected with empty adenovirus), the miR‐205 mimic group (transfected with miR‐205 mimic lentivirus), the miR‐205 inhibitor group (transfected with miR‐205 inhibitor lentivirus), the AR overexpression group (transfected with AR overexpression lentivirus) and the miR‐205 mimic +AR overexpression group (transfected with miR‐205 mimic and AR overexpression lentiviruses). All lentiviruses were constructed by Shanghai Genechem Co., Ltd. (Shanghai, China). Next, the rats were administered intraperitoneal injections with 0.4% nembutal by 35 mg/kg, and immediately fixed on fixed trays in the supine position after anaesthetization. The corpora penis was fully exposed and ligated at the root using elastic in order to obstruct injecting fluid into the systemic circulation. Then, 50 µL of virus diluent (1 × 10^7^ virus) was injected into cavernosa with an insulin syringe within 1 minute. The insulin needle was placed on the cavernosa for 5 minutes, and then extracted carefully, and a tampon was used for hemostasia. After the elastic was loosened, the rats were placed in cages for feeding. The times of penile erection, body weight and blood glucose levels of rats were recorded again.

### 2.4 Measurement of intracavernosal pressure and mean arterial blood pressure

After transfection, the rats from each group were anaesthetized, and fixed again according to the aforementioned procedures, the median skin of the neck and muscle were cut‐off, and the common carotid artery was isolated to avoid vagal nerve destruction. Next, the distal end of the isolated common carotid artery was ligated (the proximal end was not ligated), and a V‐shaped incision was carefully made through the common carotid artery under an anatomical microscope. The PE50 tube (which was flushed with heparin and connected to the PowerLab/4sp A/D converter) was rapidly inserted into the proximal suture along the incision. The vessel and PE50 tube were clamped using tweezers, and the common carotid artery and PE50 tube were fixed in order to determine the mean arterial blood pressure (MAP). Skin overlying the median abdomen was incised to isolate muscles and fascia, and to fully expose the prostate gland and carefully dissected the surrounding fat tissues. Pelvic ganglion and cavernous body nerve were found under an anatomic microscope, and the skin and fascia of the penis were cut to fully expose the penile tissues. Under the anatomic microscope, a 25‐gauge intravenous infusion needle (which was flushed with heparin and connected to the three‐way tube and PowerLab/4sp A/D converter) was carefully inserted into the right cavernous body of the penis, accompanied by 250 U/mL heparin injections to observe whether it was smooth or not (if not smooth, the needle was adjusted until it was smooth). With 15 HZ, 1.2 ms serving as the electrical stimulation parameter, a bipolar hook electrode was used to stimulate the cavernous nerve of the penis, and 5. 0 V voltage was for stimulation. The stimulation was carried out for 1 minute (three times at an interval of 10 minutes between two stimulations). The two channels were connected with the pressure transducer respectively and directly connected to a pressure transducer BL‐410 biologic function experiment system (Chengdu Taimeng Technology Co., Ltd., Chengdu, Sichuan, China), and accordingly, the MAP and intracavernosal pressure (ICP) values were recorded. The ratio of ICP to MAP was used as the evaluation standard of erectile function in rats as follows: erectile function (%) = ICP/MAP ×100%. After the measurement was carried out, blood samples were obtained from the caudal artery, and the serum content was separated. In addition, radioimmunoassay was employed in order to determine the serum testosterone levels. Then, the rats were sacrificed with excessive pentobarbital sodium. The fascia surrounding the penile tissues was rapidly dissociated, and the whole penis (including the cavernosum) was cut‐off and rinsed two times with phosphate buffer saline (PBS). The cavernosa tissues were fixed with 4% paraformaldehyde overnight, embedded in paraffin and sectioned continuously. The remaining corpus cavernosa tissues were placed in a cryopreserved tube, and preserved in liquid nitrogen pot at −80℃. Referring to the quantitative real‐time polymerase chain reaction (qRT‐PCR) procedure of cell experiment, the expression of miR‐205 and AR in the corpus cavernosum of normal rats and DMED rats was detected. Each experiment was repeated three times to obtain the mean value.

### 2.5 Hematoxylin and eosin staining

Cavernosa tissues were fixed in 4% formaldehyde for 24 hours, washed under tap water and sliced into 5‐μm‐thick sections after convention dehydration, clearing two times with xylene (5 minutes each time) and cooled on the cold table of the paraffin‐embedded machine. Next, the paraffin sections were heated for 1 hour at 70°C, and then heated for 5 hours at 60°C. Then, the sections were dewaxed, stained for 10 minutes using haematoxylin (PT001, Shanghai Bogoo Co., Ltd., Shanghai, China), washed for 30‐60 seconds under running water, differentiated for 30 seconds in 1% ethanol hydrochloride, rinsed for 5 minutes under running water again, and then stained with eosin (Beijing XinHuaLvYuan Science and Technology Ltd., Beijing, China) for 1 minute at room temperature. After that, the sections were soaked with ethanol for dehydration. Next, the sections were cleared with xylene carbolic acid for 1 minute and two times with xylene I and II (Shanghai Guduo Biotechnology Co., Ltd., Shanghai, China) respectively, 1 minute each time, and sealed with resinene. Finally, the morphology of cavernosa tissues was observed under a Zeiss fluorescence microscope (PrimoStariLED, Beijing Boruisi Technology Co., Ltd., Beijing, China).

### 2.6 Masson staining

The paraffin sections were heated at 65°C for 3 hours, dewaxed and dehydrated conventionally, rinsed for 40 minutes in 10% trichloroacetic acid and 10% kalium dichromicum, and washed under tap water. Next, the sections were stained using haematoxylin (Shanghai Bogoo Co., Ltd., Shanghai, China) for 8 minutes and washed under tap water. Then, the sections were rinsed using a mixture of 1% ponceau (HL12202, Shanghai Haling Biotechnology Co., Ltd., Shanghai, China) and 1% magenta (HPBIO‐SJ820, Hepeng Biotechnology Co., Ltd., Shanghai, China). The reaction was terminated with 1% ice vinegar and 1% molybdic acid, followed by treatment of 1% brilliant green, 1% phosphomolybdic acid and tap water. Subsequently, conventional dehydration, clearing and mounting with resinene were carried out. Twenty high‐power visual fields of each slice were randomly selected, and the staining results of smooth muscle and connective tissue were observed under an optical microscope.

### 2.7 Immunohistochemistry

Paraformaldehyde‐fixed cavernosa tissues were dehydrated, cleared, embedded in paraffin and sliced into 4‐μm‐thick sections. The sections were supplemented with 10% normal goat serum (C‐0005, Shanghai Haoran Bio Technologies Co., Ltd., Shanghai, China), incubated at room temperature for 20 minutes. Next, the sections were incubated with the addition of primary rabbit anti‐rat antibody to AR (dilution ratio of 1:200, Abcam Inc, Cambridge, MA, USA) overnight at 4°C. Then, the sections were rinsed three times with 0.1 M PBS (5 minutes per rinse), incubated with the drop of secondary goat anti‐rabbit antibody to IgG (dilution ratio of 1:1000, Abcam Inc, Cambridge, MA, USA) for 20 minutes at 37°C. After that, the sections were rinsed three times with 0.1 M PBS (5 minutes per rinse), coloured with diaminobenzidine (DAB, ST033, Guangzhou Whiga Company, Guangzhou, Guangdong, China) for 5‐10 minutes. After being washed, the sections were counterstained with haematoxylin (PT001, Shanghai Bogoo Co., Ltd., Shanghai, China) for 1 minute and then rinsed with water. Finally, the sections were soaked with 1% ammonia water for 10 seconds, washed and sealed using resinene. Subsequently, the sections were observed under a 100‐fold microscope with PBS instead of the primary antibody serving as the negative control. A total of five high‐power visual fields with 100 cells in each field were randomly selected and observed. The positive cells were identified as those with staining degree greater than 25% with brown or tan granules in the nucleus or cytoplasm.^19^ When the positive area was located, the image was obtained using a colour camera, and input in the ‘Video Pro32’ colour image analysis system. The grey value of the area was measured after the positive area was segmented accurately.^20^

### 2.8 Terminal deoxynucleotidyl transferase (TdT)‐mediated dUTPbiotin nick end labelling (TUNEL) assay

Cavernosa tissues were fixed in 4% paraformaldehyde, embedded in paraffin and sectioned. According to the instructions of the TUNEL kit (40302ES20, Yeasen Biotechnology Co. Ltd., Shanghai, China), the sections were dewaxed conventionally, rinsed with PBS for 10 minutes, and then incubated with the addition of 50 μL of blocking solution containing 0.3% H_2_O_2_ in methanol for 10 minutes after drying the liquid around the sample. After being rinsed with PBS for 10 minutes, the sections were ice‐bathed in penetrant for 2 minutes and re‐rinsed three times with PBS (10 minutes per rinse). Next, the sections were cultured with 50 μL of TUNEL reaction solution at 37°C for 1 hour in a wet box, and rinsed three times with PBS (10 minutes per rinse). Next, the negative staining control was added with the TdT‐free reaction solution.^21^ Alkaline phosphatase‐labelled antibodies (BAR301, Beijing Berseebio Co., Ltd., Beijing, China) were added, and 50 μL of DAB (Guangzhou Whiga Biotechnology, Guangzhou, China) was used for developing for 10 minutes. Then, the samples were rinsed three times with PBS (10 minutes per rinse), and stained with haematoxylin (Shanghia Affandi Company, Shanghai, China), followed by gradient ethanol dehydration, xylene clearing and resinene sealing. The apoptotic cells presented tan coloration in nucleus, and normal cells presented blue coloration in nucleus. A digital pathology slice scanner (6504523001, Roche Diagnostics Corp., Shanghai, China) was used for scanning the sections. A total of five visual fields of each section were randomly selected and observed (using 400 × magnification). The apoptotic index was exhibited as the mean of the ratio of the positive cell number to the total cell number.

### 2.9 Quantitative real‐time polymerase chain reaction

Total RNA content of cavernosa tissues from each group was extracted in strict accordance with the instructions of the Trizol kit (15596‐018, Beijing Solarbio Science & Technology Co., Ltd., Beijing, China), and subsequently, its concentration was measured. All primers employed in the current study were synthesized by Takara Biotechnology Ltd. (Dalian, Liaoning, China) and shown in Table 1. Reverse transcription was performed using a cDNA reverse transcription system (K1622, Beijing Reanta Company, Beijing, China), with the reaction condition set as 30‐50 minutes at 42°C and 5 seconds at 85°C. The obtained cDNA was diluted to 50 ng/μL, and 2 μL was used each time for 25 μL of amplification system. qRT‐PCR was then performed with 2 μg cDNA serving as the templet using a fluorescence quantitative PCR instrument (ViiA 7, Daan Gene Co., Ltd., Guangzhou, Guangdong, China). Reaction conditions were as follows: pre‐denaturation at 95°C for 4 minutes, a total of 30 cycles of denaturation at 95°C for 30 seconds, annealing at 57°C for 30 seconds and extension at 72°C for 30 seconds. U6 was regarded as the internal reference for miR‐205, and β‐actin served as the internal reference for AR, Caspase‐3, Bax, and Bcl‐2. The 2^‐ΔΔCt^ method was employed in order to compare relative mRNA expressions, and the formula was as follows: ΔΔCt = ΔCt _the model group _‐ ΔCt _the normal group_, in which ΔCt = Ct _target gene_ ‐ Ct _internal reference_. The relative mRNA expression of target genes was equal to the value of ΔΔCt 22. The experiment was repeated three times to obtain the mean value. The aforementioned method was also applicable for cell experiments.

**Table 1:** The primer sequences for qRT‐PCR

Gene	Sequence (5'‐3')
miR‐205	Forward: GGGACCAACAAACCACCGGA
	Reverse: CAGTGCGTGTCGTGGAGT
AR	Forward: CAGTAGCCCAAGCGATGC
	Reverse: AACTCCACCAGGATACCACA
Caspase‐3	Forward: AGAGCTGGACTGCGGTATTGAG
	Reverse: GAACCATGACCCGTCCCTTG
Bax	Forward: ACACCTGAGCTGACCTTGGA
	Reverse: CCGTGTCCACGTCAGCAATC
Bcl‐2	Forward: CGGGAGAACAGGGTATGA
	Reverse: CAGGCTGGAAGGAGAAGAT
U6	Forward: GCTCGCTTCGGCAGCACA
	Reverse: GAGGTATTCGCACCAGAGGA
β‐actin	Forward: GGAGATTACTGCCCTGGCTCCTA
	Reverse: GACTCATCGTACTCCTGCTTGCTG

AR, androgen receptor; Bax, BCL2‐associated X protein; Bcl‐2, B cell leukaemia/lymphoma 2; qRT‐PCR, quantitative real‐time polymerase chain reaction.

### 2.10 Western blot analysis

Extracted cavernosa tissues from each group were treated with protein lysis at 4°C for 30 minutes, vibrated once every 10 minutes, and then centrifuged at 2000 × *g* for 20 minutes at 4°C. The fat layer was discarded, and the supernatant was collected as the protein extract. Total protein concentration was measured using a bicinchoninic acid kit (20201ES76, Shanghai Yeasen Biotechnology Co., Ltd., Shanghai, China). Next, quantitation experiment was performed based on different concentrations. Briefly, the protein was separated using polyacrylamide gel, transferred onto polyvinylidene fluoride membranes and then blocked with 5% bovine serum albumin in room temperature for 1 h. The membrane was incubated with the addition of primary rabbit anti‐rat antibodies to AR (ab74272, dilution ratio of 1:1000), Caspase‐3 (AC033, dilution ratio of 1:500), Bax (ab32503, dilution ratio of 1:5000) and Bcl‐2 (ab59348, dilution ratio of 1:800) overnight. All aforementioned antibodies were provided by Abcam Inc (Cambridge, MA, USA). After being rinsed three times in Tris‐buffered saline plus 0.1% Tween 20 (TBST) (5 minutes per rinse), the membrane was incubated at room temperature for 1 hour with the horseradish peroxidase‐labelled secondary goat anti‐rabbit antibody to IgG (ab205718, dilution ratio of 1:20000, Abcam Inc, Cambridge, MA, USA). After that, the membrane was re‐rinsed three times with TBST (5 minutes per rinse), and added with an electro‐chemiluminescence (Pierce, Waltham, MA, USA) developer. Quantitative protein analysis was conducted by comparing the ratio of targeted grey values to internal reference gene glyceraldehyde‐3‐phosphate dehydrogenase using the Image J 1.48u software (National Institutes of Health, Bethesda, MD, USA). The experiment was repeated three times to obtain the mean value.

### 2.11 Cell culture and transfection

Cavernous smooth muscle cells (CSMCs) of the penis were cultured in a humidified incubator using the attachment‐block method with Royal Park Memorial Institute (RPMI) 1640 medium (Gibco, Gaithersburg, MD, USA) containing 10% foetal bovine serum (FBS, Hyclone, Logan, UT, USA) at 37°C with 5% CO_2 _in air. After being treated with 0.25% trypsin (Gibco, Gaithersburg, MD, USA), the cells were triturated into a single cell suspension using the RPMI 1640 medium containing 10% FBS, and then were sub‐cultured conventionally. Next, the cells in the logarithmic phase of growth were collected for further experimentation.

Subsequently, the CSMCs were divided into various groups, namely, the control group, the NC group (transfected with empty adenovirus), the miR‐205 mimic group (transfected with miR‐205 mimic lentivirus), the miR‐205 inhibitor group (transfected with miR‐205 inhibitor lentivirus), the AR overexpression group (transfected with AR overexpression lentivirus), and the miR‐205 mimic +AR overexpression group (transfected with miR‐205 mimic and AR overexpression lentivirus). All aforementioned lentiviruses were purchased from Shanghai Genechem Co., Ltd. (Shanghai, China). CSMCs in the logarithmic phase of growth were seeded into a six‐well plate until the cell density reached 30%‐50%. Cell transfection was carried out using the protocol of lipofectamine 2000 (Invitrogen Inc, Carlsbad, CA, USA). Briefly, 100 pmol cells in the NC, miR‐205 mimic, miR‐205 inhibitor, AR overexpression and miR‐205 mimic +AR overexpression groups were diluted with 250 µL of serum‐free Opti‐MEM (Gibco, Gaithersburg, MD, USA) with a final concentration of 50 nM, and then incubated for 5 minutes at room temperature. Next, 5 µL of lipofectamine 2000 was diluted with 250 µL of serum‐free Opti‐MEM, and incubated for 5 minutes at room temperature. The above two products were mixed, incubated at room temperature for 20 minutes, and placed in cell culture plates. Complete medium was used to replace the old medium after 6‐8 hours of incubation at 37°C in 5% CO_2_. The follow‐up experiment was carried out after 24‐48 hours of incubation.

### 2.12 Dual‐luciferase reporter gene assay

The target relationship between miR‐205 and AR was predicted using a bioinformatics website (https://cm.jefferson.edu/rna22/Interactive/), and a luciferase reporter gene assay was carried out in order to further verify whether AR is a target gene of miR‐205. Target sequences and mutation sequences were designed in accordance with the binding sequence of AR‐3’‐untranslated region (UTR) and miR‐205. Simultaneously, the two sides of sequences were complemented with endonuclease sites Xho I and Xho I respectively. The target fragment was inserted into the PUC57 vector (HZ0087, Zhen Shanghai and Shanghai Industrial Co., Ltd., Shanghai, China). After identification of positive clones, the recombinant plasmids were identified by DNA sequencing, sub‐cloned into the psiCHECK‐2 vector (HZ0197, Zhen Shanghai and Shanghai Industrial Co., Ltd., Shanghai, China), and transformed into *Escherichia coli* DH5α cells in order to amplify the plasmids. Then the plasmids were extracted according to the instructions of the Omega kit (D1100‐50 T, Beijing Solarbio Science & Technology Co., Ltd., Beijing, China). After extraction, the cells were inoculated into a six‐well plate with a density of 2 × 10^5^ cells/well. The cells were transfected according to the instructions of lipofectamine 2000 after being observed to be adhered to the plates. The cells were harvested at the 48th hour of culture after transfection. The effect of miR‐205 on the luciferase activity of AR 3’‐UTR in cells was examined according to the instructions provided by the dual‐luciferase assay kit (D0010, Beijing Solarbio Science & Technology Co., Ltd., Beijing, China). The fluorescence intensity was measured using a Glomax 20/20 luminometer fluorescence detector (E5311, Shanxi Zhongmei Biotechnology Co., Ltd., Shanxi, China).

### 2.13 Pull‐down assay

Cavernous smooth muscle cells were transfected with wide‐type (WT) biotinylated miR‐205 (50 nM, Bio‐miR‐205‐WT) and mutant (MUT) biotinylated miR‐205 (50 nM, Bio‐miR‐205‐MUT). After transfection for 48 hours, the cells were collected, rinsed with PBS, incubated for 10 minutes with specific cell lysates (Ambion, Austin, Texas, USA) and sub‐packed at a volume of 50 mL. Next, the remnant was cultured at 4°C for 3 hours with streptavidin‐biotin and magnetic beads (M‐280) pre‐coated with RNase‐free and yeast tRNA (Sigma‐Aldrich Chemical Company, St Louis, MO, USA), rinsed two times with cold lysate, rinsed three times with low‐salt buffer and rinsed once with high‐salt buffer. The miR‐205 antagonist probe served as the negative control. Total RNA content was extracted using Trizol, and the expression of AR was determined by qRT‐PCR.

### 2.14 3‐(4, 5‐dimethyl‐2‐thiazolyl)‐2, 5‐diphenyl‐2‐H‐tetrazolium bromide (MTT) assay

After transfection for 48‐hours, CSMCs from each group were collected and counted, then seeded into a 96‐well plate at a density of 6 × 10^3^ cells/well with 100 μL medium in each well, and cultured in a humidified incubator with six parallels set up. Three time intervals, that is 24, 48 and 72 hours were set for the following experiments. Each well was added with 20 μL of MTT solution (5 mg/mL), and incubated for 2 hours at 37°C. After the supernatants were discarded, 150 μL of dimethyl sulfoxide solution was added to each well. Subsequently, the optical density (OD) value at 570 nm of each well was measured using a microplate reader (NYW‐96 M, Beijing Nyaw Co., Ltd., Beijing, China). The cell viability curve was plotted with various time intervals (24, 48, and 72 hours) as the abscissa, and the OD values as the ordinate.

### 2.15 Flow cytometry

After 48‐hours transfection, CSMCs from each group were treated with ethylene diamine tetraacetic acid‐free trypsin, collected into flow tubes, and rinsed three times with cold PBS. According to the instructions of the Annexin‐V‐fluorescein isothiocyanate (FITC) apoptosis kit (Shanghai Yeasen Biotechnology Co., Ltd., Shanghai, China), Annexin‐V‐FITC, propidiom iodide (PI), and N‐2‐hydroxyethypiperazine‐N'‐2‐ethanesulfonic Acid (HEPES) were mixed at a ratio of 1:2:50 in order to prepare the Annexin‐V‐FITC/PI staining solution. Staining solution (100 μL) was used to re‐suspend 1 × 10^6^ cells. Next, the mixture was incubated for 15 minutes at room temperature, added with 1 mL of HEPES and mixed. Subsequently, the cells were excited at 488 nm, and FITC fluorescence was detected using a 520 nm band‐pass filter, while PI was detected using a 610 nm band‐pass filter.

### 2.16 Statistical analysis

Statistical analyses were conducted using the SPSS 21.0 software (IBM Corp. Armonk, NY, USA). Measurement data were presented as mean values ± SD. The one‐way analysis of variance (ANOVA) was used to compare values among groups, followed by the Student's *t*test to compare values between groups. The Pearson correlation analysis was carried out for the analysis of the correlation of two variable quantities. *P* < 0.05 indicated that the difference was statistically significant.

## 3 RESULTS

### 3.1 Successful establishment of DMED rat models

**Figure 1:**
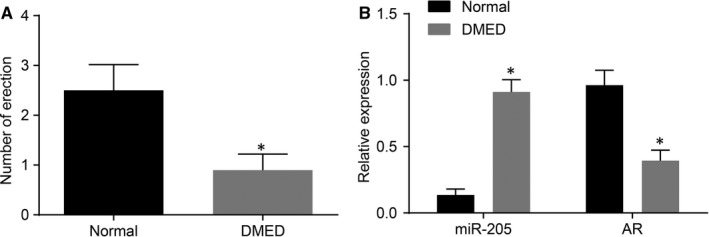
The DMED rat models are successfully established. A, the times of erection; B, the mRNA levels of miR‐205 and AR measured by qRT‐PCR; *, *P* < 0.05 vs the normal group; measurement data were expressed as mean ± SD and analysed by unpaired *t* test; the sample size of each group was 10; the experiment was repeated three times; DMED, erectile dysfunction rats with diabetic mellitus; miR‐205, microRNA‐205; AR, androgen receptor; qRT‐PCR, quantitative real‐time polymerase chain reaction.

A total of 110 rats were included in the current study, and five rats died; and nine rats presented with blood glucose levels were not more than 16.7 mmol/L during DM model establishment. The remaining 96 rats were classified as diabetic rats, exhibiting blood glucose levels greater than 16.7 mmol/L. Diabetic rats generally presented with symptoms of mental depression, weight loss, polydipsia, polyphagia, polyuria, dark and dull hair. After APO induction, diabetic rats exhibited hair erection and yawning. Among the included 96 rats, 70 rats exhibiting no penile erection after 30 minutes of APO administration were diagnosed as ED, indicating a modelling success rate of 72.91%. The times of erection stimulated by APO after 1 week of modelling were recorded, and the results showed (Figure 1A) that the times of erection of rats with DMED were significantly reduced compared with the normal rats. In addition, the expression of miR‐205 and AR in cavernosa tissues of rats with DMED and normal rats using qRT‐PCR. Interestingly, the expression of miR‐205 in rats with DMED was found to be significantly higher than those in the normal rats, while the expressions of AR mRNA were significantly lower respectively (both *P* < 0.05) (Figure 1B). The above results indicated that rats suffering from DMED exhibited high expression of miR‐205, in addition to reduced AR expressions.

### 3.2 MiR‐205 down‐regulation or AR up‐regulation improves the erectile functioning of rats with DMED

Body weight and blood glucose level changes are shown in Table 2. After STZ administration, the body weight and blood glucose level were found to be dramatically elevated (*P* < 0.05). There were no significant differences in the body weight and blood glucose levels among the NC, DMED and miR‐205 mimic +AR overexpression groups (*P* > 0.05). In comparison with the DMED group and the NC group, the body weight and the blood glucose levels of rats in the miR‐205 mimic group were found to be significantly increased after STZ administration respectively (both *P* < 0.05); whereas the body weight and the blood glucose levels of rats in the miR‐205 inhibitor group and the AR overexpression group were decreased (both *P* < 0.05).

**Table 2:** STZ‐inducement leads to statistically significant difference in body weight and blood sugar level of rats

Group	Number	Weight (g)		Blood glucose level (mmol/L)	
		Before STZ induction	After STZ induction	Before STZ induction	After STZ induction
DMED	10	248.62 ± 18.54	258.38 ± 13.64	6.51 ± 0.61	24.73 ± 2.58
NC	10	249.88 ± 15.64	262.37 ± 14.92	6.62 ± 0.48	23.96 ± 1.88
miR‐205 mimic	10	246.27 ± 16.45	312.21 ± 13.57^a^^,^ b	6.44 ± 0.52	32.78 ± 2.71^a^^,^ b
miR‐205 inhibitor	10	251.34 ± 17.46	202.45 ± 17.42^a^^,^ b	6.41 ± 0.63	15.69 ± 1.68^a^^,^ b
AR overexpression	10	252.39 ± 16.82	205.76 ± 14.63^a^^,^ b	6.67 ± 0.54	14.88 ± 1.86^a^^,^ b
miR‐205 mimic +AR overexpression	10	249.72 ± 15.41	265.74 ± 15.25	6.56 ± 0.63	24.99 ± 2.37

AR, androgen receptor; DMED, erectile dysfunction rats with diabetic mellitus; miR‐205, microRNA‐205; STZ, streptozocin.

Measurement data was expressed as mean ± SD; data in each group were analysed with ANOVA; the sample size of each group was 10; the experiment was repeated three times.

a *P *< 0.05 vs. the normal group.

b *P *< 0.05 vs. the DMED group and the NC group.

**Figure 2:**
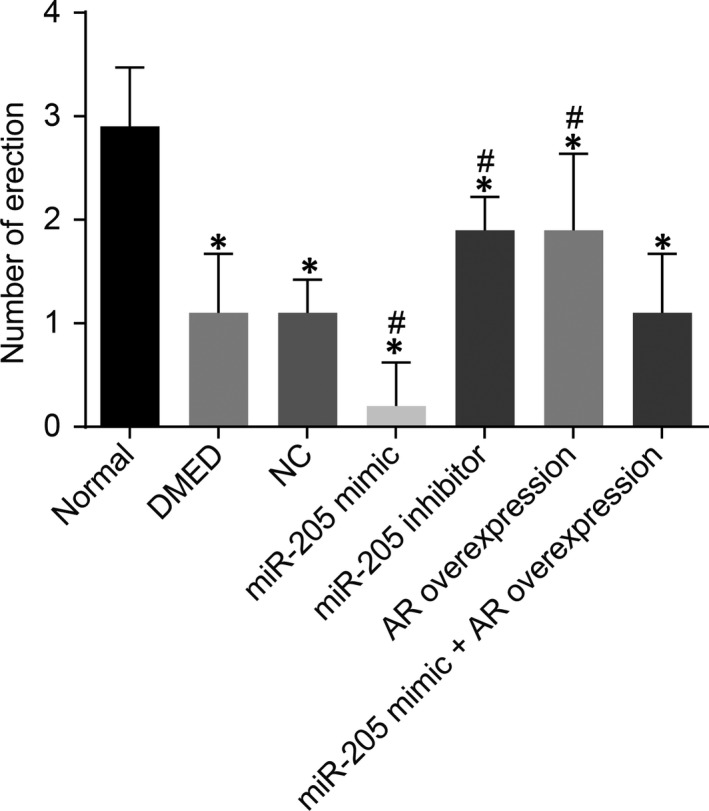
miR‐205 down‐regulation enhances the times of erection in rats with DMED. *, *P* < 0.05 vs the normal group; #, *P* < 0.05 vs the DMED group and the NC group; measurement data were expressed as mean ± SD; data in each group were analysed with ANOVA; the sample size of each group was 10; the experiment was repeated three times; DMED, erectile dysfunction rats with diabetic mellitus; miR‐205, microRNA‐205; AR, androgen receptor; NC, negative control.

In comparison with the normal group, decreased times of erection of rats were recorded in all other groups (*P* < 0.05). There were no significant differences in erection times among the NC, DMED and miR‐205 mimic +AR overexpression groups (*P* > 0.05). Compared with the DMED group and the NC group, the times of erection of rats in the miR‐205 mimic group were found to be markedly reduced, while those in the miR‐205 inhibitor group and the AR overexpression group were significantly increased (*P < *0.05) (Figure 2).

The results of electrical stimulation testing are shown in Table 3 as well as in the Figures S1 and S2. The MAP, ICP and ICP/MAP values were found to be significantly decreased in rats in the DMED, NC, miR‐205 mimic, miR‐205 inhibitor, AR overexpression and miR‐205 mimic +AR overexpression groups in comparison with the normal group (all *P* < 0.05). There were no significant differences in the MAP, ICP and ICP/MAP values among the NC, DMED and miR‐205 mimic +AR overexpression groups (all *P* > 0.05). Compared with the DMED group and the NC group, the MAP, ICP and ICP/MAP values in the miR‐205 mimic group were significantly decreased (all *P* < 0.05), while the MAP, ICP and ICP/MAP values of the miR‐205 inhibitor group and the AR overexpression group were significantly increased (all *P* < 0.05).

**Table 3:** Down‐regulation of miR‐205 and up‐regulation of AR expression decrease the MAP, ICP and ICP/MAP value

Group	Number	MAP (mm Hg)	ICP (mm Hg)	ICP/MAP
Normal	10	112.93 ± 10.28	98.45 ± 9.82	0.88 ± 0.12
DMED	10	89.32 ± 7.45^a^	27.46 ± 3.41^a^	0.31 ± 0.06^a^
NC	10	91.62 ± 8.69^a^	26.75 ± 2.61^a^	0.3 ± 0.03^a^
miR‐205 mimic	10	89.57 ± 7.26^a^, ^b^	19.68 ± 0.83^a^, ^b^	0.22 ± 0.02^a^, ^b^
miR‐205 inhibitor	10	129.49 ± 9.65^a^, ^b^	62.34 ± 5.66^a^, ^b^	0.49 ± 0.07^a^, ^b^
AR overexpression	10	132.81 ± 10.73^a^, ^b^	61.62 ± 6.52^a^, ^b^	0.46 ± 0.03^a^, ^b^
miR‐205 mimic +AR overexpression	10	87.46 ± 8.28^a^	26.98 ± 2.46^a^	0.31 ± 0.04^a^

AR, androgen receptor; DMED, erectile dysfunction rats with diabetic mellitus; miR‐205, microRNA‐205; NC, negative control.

The level of MAP, ICP and ICP/MAP were measurement data, which was expressed as mean ± SD; data in each group was analysed with ANOVA; the sample size of each group was 10; the experiment was repeated for three times.

a *P* < 0.05 vs. the normal group.

b *P *< 0.05 vs. the DMED group and the NC group.

After ICP/MAP recording and prior to rat sacrifice, blood samples were obtained from the caudal artery, and serum was isolated. In addition, serum testosterone concentration was measured using radioimmunoassay. Results as shown in Table S1 demonstrate that the serum testosterone concentration of rats in each group was consistent with that of electrical stimulation, indicating that testosterone concentration was directly correlated to the progression of ED. The findings also indicate that testosterone and AR may be involved in the influence of DM exerted on penile erectile functioning. These results suggested that the down‐regulation of miR‐205 or overexpression of AR might improve erectile functioning in rats with DMED.

### 3.3 MiR‐205 down‐regulation or AR overexpression alleviates the pathology of cavernous bodies

**Figure 3:**
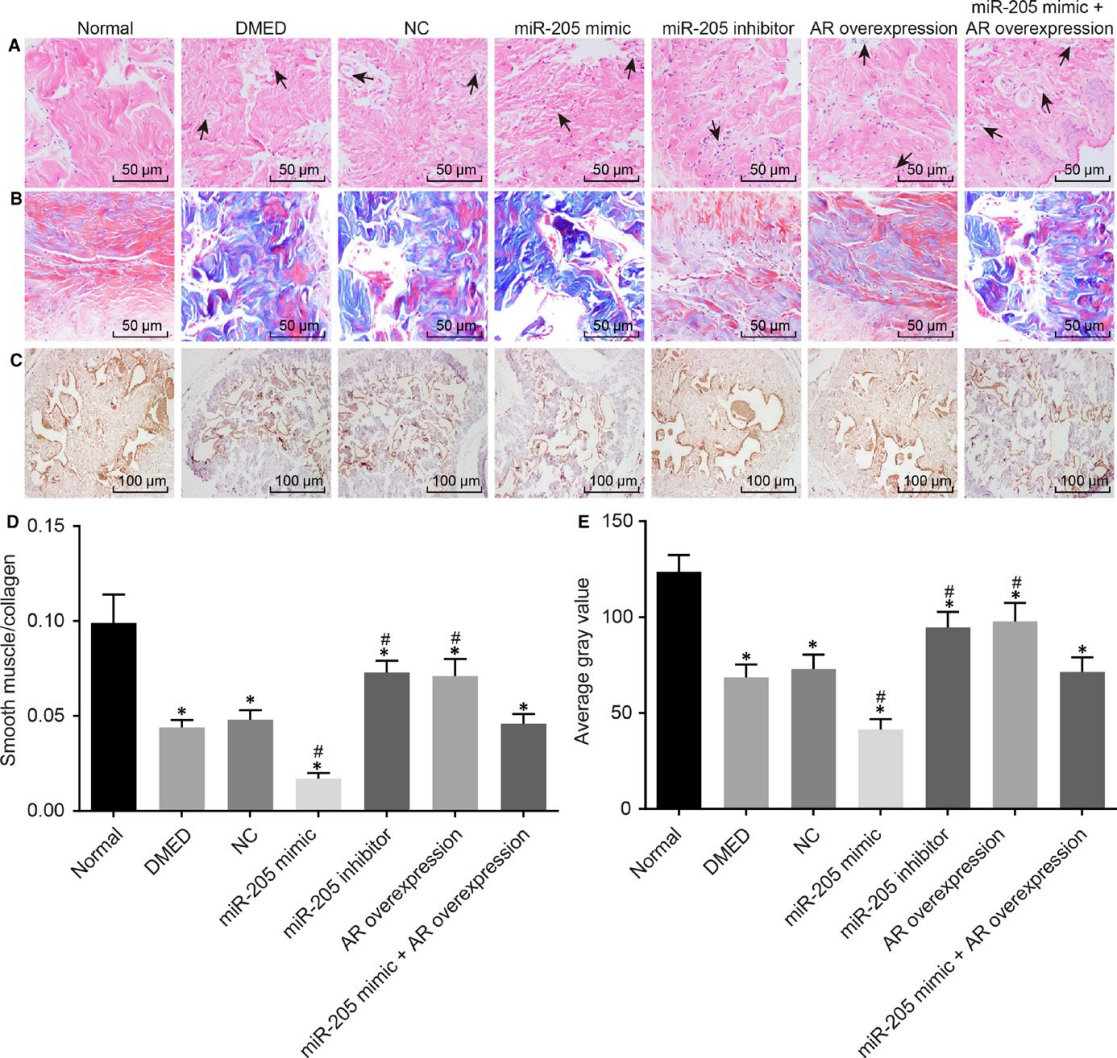
Down‐regulation of miR‐205 or overexpression of AR could prevent the pathological progression of DMED and inhibit the fibrosis of corpus cavernosum. A, pathological changes of cavernous bodies of rats treated with miR‐205 mimic, miR‐205 inhibitor, AR overexpression, and miR‐205 mimic +AR overexpression were observed by hematoxylin and eosin staining with the arrow indicating the diseased tissue (200 ×), the arrow indicates the diseased tissue; B, smooth muscle density in cavernous bodies of rats treated with miR‐205 mimic, miR‐205 inhibitor, AR overexpression, and miR‐205 mimic +AR overexpression was observed by Masson staining (400 ×); C, Immunohistochemical staining was applied to compare the positive expression rate of AR in the cavernous bodies of rats treated with miR‐205 mimic, miR‐205 inhibitor, AR overexpression, and miR‐205 mimic +AR overexpression (200 ×); D, statistical analysis of smooth muscle density measured by Masson staining; E, statistical analysis of AR positive expression in the cavernous bodies of rats treated with miR‐205 mimic, miR‐205 inhibitor, AR overexpression and miR‐205 mimic +AR overexpression; *, *P* < 0.05 vs the normal group; #, *P* < 0.05 vs the DMED group and the NC group; measurement data were expressed as mean ± SD; data in each group were analysed with ANOVA; the sample size of each group was 10; the experiment was repeated three times; DMED, erectile dysfunction rats with diabetic mellitus; miR‐205, microRNA‐205; AR, androgen receptor; NC, negative control.

In the following experiments, the current study evaluated the effects of miR‐205 and AR on the pathology of cavernous bodies. A large number of new capillaries were observed in the corpus cavernosum tissues of rats of all groups but not in the normal group (Figure 3A). In comparison with the DMED group and the NC group, the number of new capillaries in the corpus cavernosum tissues in rats of the miR‐205 mimic group was observed to be significantly increased, while rats of the miR‐205 inhibitor group and the AR overexpression group presented with significantly decreased numbers. These results suggest that down‐regulation of miR‐205 or up‐regulation of AR expression might improve the pathological changes of cavernous bodies of the penis by diminishing the number of new capillaries in the corpus cavernosum tissues.

Changes of smooth muscle density in the cavernosa were evaluated by the ratio of red positive area to total area with Masson staining. In the normal group, a large number of cavernous smooth cells stained with red were observed in the corpus cavernosum of penis, and the fibres of the smooth muscle were clearly visible and neatly arranged. A great amount of blue‐stained connective tissues were observed in the corpus cavernosum in rats of all groups but not in the normal group, and the smooth muscle density of the corpus cavernosum was decreased and the fibres of smooth muscle were in disorderly arrangement. In comparison with the DMED group and the NC group, red‐stained CSMCs were hardly observed, and the smooth muscle density of the corpus cavernosum was decreased in the miR‐205 mimic group; the smooth muscle density of the corpus cavernosum was increased in the miR‐205 inhibitor group and the AR overexpression group (Figure 3B,D). Simultaneously, the positive expression rate of AR was measured using immunohistochemistry. AR was found to be positively expressed in the nucleus of cells. The grey values of AR positive expression were significantly diminished in all groups but not in the normal group (all *P* < 0.05). There were no significant differences in the positive expressions of AR among the NC, DMED and miR‐205 mimic +AR overexpression groups (*P* > 0.05). Compared with the DMED group and the NC group, the AR positive expression in the miR‐205 mimic group was reduced, while the AR positive expression was significantly elevated in the miR‐205 inhibitor group and the AR overexpression group (both *P* < 0.05) (Figure 3C,E). These results suggested that down‐regulation of miR‐205 or up‐regulation of AR could prevent tissue fibrosis in the corpus cavernosum in rats with DEMD.

### 3.4 MiR‐205 down‐regulation or AR overexpression inhibits apoptosis of CSMCs in rats with DMED

**Figure 4:**
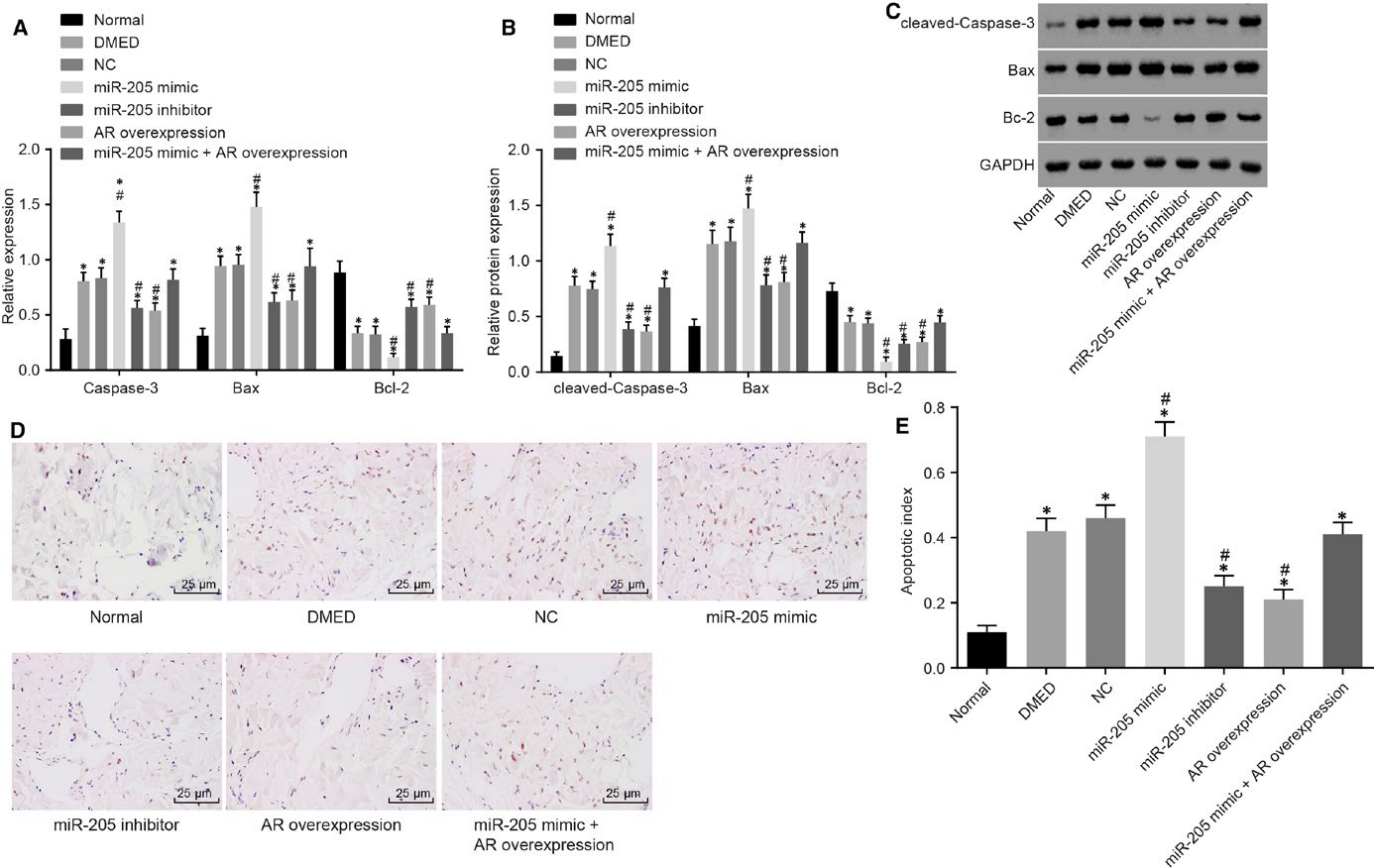
The miR‐205 down‐regulation leads to the decreased cell apoptosis in the cavernous bodies of rats with DMED. A, the mRNA levels of Caspase‐3, Bax and Bcl‐2 in cavernous bodies of rats treated with miR‐205 mimic, miR‐205 inhibitor, AR overexpression, and miR‐205 mimic +AR overexpression determined by qRT‐PCR; B, the protein levels of Caspase‐3, Bax and Bcl‐2 in cavernous bodies of rats treated with miR‐205 mimic, miR‐205 inhibitor, AR overexpression, and miR‐205 mimic +AR overexpression determined by Western blot analysis; C, the grey value of Caspase‐3, Bax and Bcl‐2 protein in cavernous bodies of rats treated with miR‐205 mimic, miR‐205 inhibitor, AR overexpression, and miR‐205 mimic +AR overexpression determined by Western blot analysis; D, TUNEL staining reflecting apoptotic cells in the cavernous bodies (400 ×); E, apoptosis rate of cells in the cavernous bodies; *, *P* < 0.05 vs the normal group; #, *P* < 0.05 vs the DMED group and the NC group; measurement data were expressed as mean ± SD; data in each group were analysed with ANOVA; the experiment was repeated three times; DMED, erectile dysfunction rats with diabetic mellitus; miR‐205, microRNA‐205; AR, androgen receptor; NC, negative control.

QRT‐PCR and Western blot analysis were employed to determine the expression of apoptosis‐related genes, including Caspase‐3, Bax and Bcl‐2 in order to explore the mechanism of down‐regulation of miR‐205 and up‐regulation of AR in apoptosis of CSMCs in rats with DMED. Significantly increased Caspase‐3 and Bax expressions in addition to significantly decreased Bcl‐3 expressions were found in all groups in comparison with the normal group (all *P* < 0.05, Figure 4A‐C). There were no significant differences in the expressions of Caspase‐3, Bax and Bcl‐2 among the NC, DMED and miR‐205 mimic +AR overexpression groups (*P* > 0.05). Compared with the DMED group and the NC group, the expressions of Caspase‐3 and Bax in the miR‐205 mimic group were significantly increased, while the expressions of Bcl‐2 were decreased; the expressions of Caspase‐3 and Bax were significantly decreased in the miR‐205 inhibitor group and the AR overexpression group, in addition to increased Bcl‐2 expressions (all *P* < 0.05). Moreover, AR overexpression could block miR‐205 mimic‐induced Bax up‐regulation and Bcl‐2 decline.

Additionally, TUNEL assay was performed in order to measure the apoptosis of CSMCs. The results showed a significant increase in apoptosis of CSMCs in all groups compared with the normal group (all *P* < 0.05). There were no significant differences in cell apoptosis rates among the NC, DMED and miR‐205 mimic +AR overexpression groups (*P* > 0.05). Compared with the DMED group and the NC group, cell apoptosis of cells in the miR‐205 mimic group exhibited a further significant increase, while there was a decrease in the miR‐205 inhibitor group and the AR overexpression group (all *P* < 0.05) (Figure 4D,E), suggesting that down‐regulation of miR‐205 and up‐regulation of AR might inhibit the apoptosis of CSMCs in rats with DMED.

### 3.5 MiR‐205 targets and negatively regulates AR

**Figure 5:**
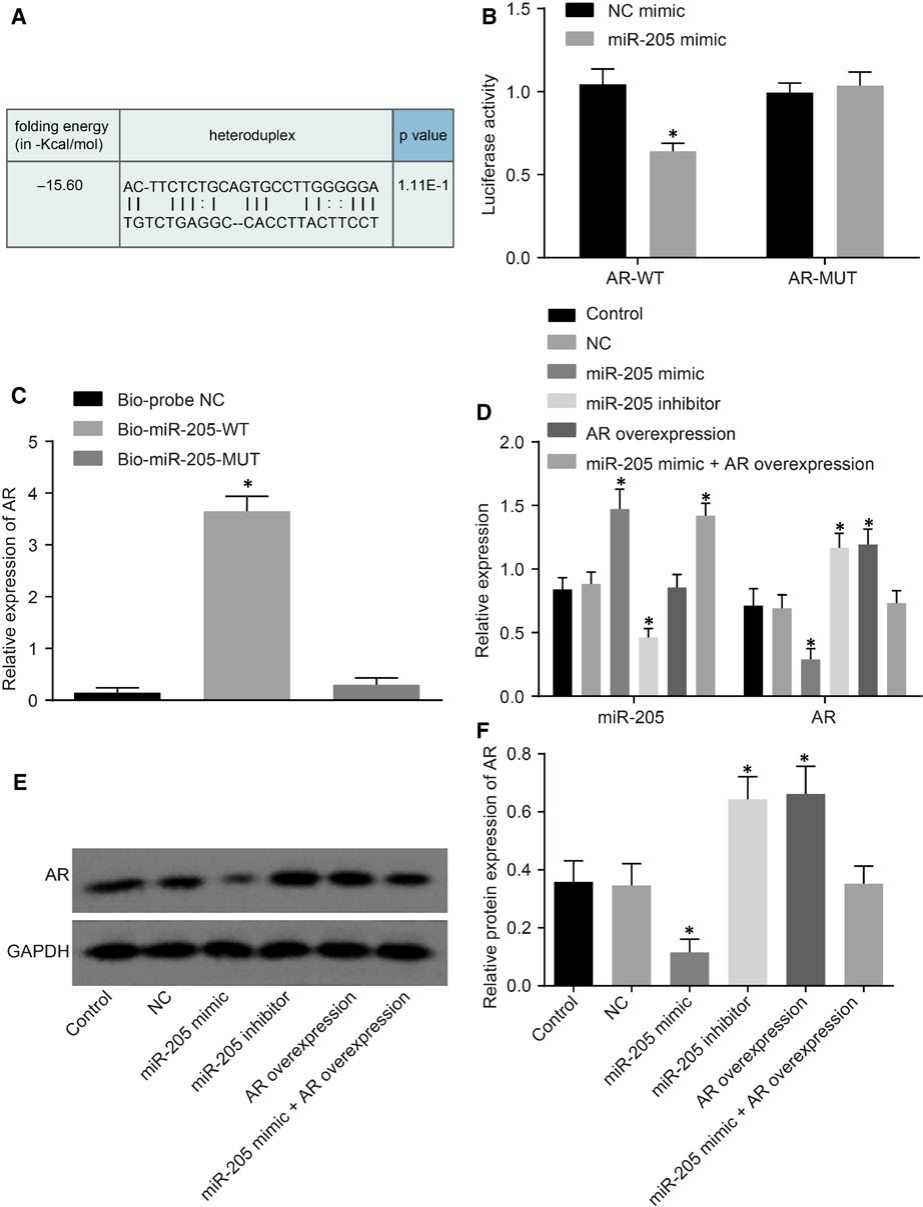
AR is a target gene of miR‐205. A, binding regions between AR 3'‐UTR and miR‐205 sequence; B, luciferase activity of the AR Wt and AR Mut after transfection; C, RNA pull‐down results showed that AR binds to miR‐205; D, the expression of miR‐205 and AR in cells treated with miR‐205 mimic, miR‐205 inhibitor, AR overexpression, and miR‐205 mimic +AR overexpression determined by qRT‐PCR; E, the grey value of miR‐205 and AR protein bands in cells treated with miR‐205 mimic, miR‐205 inhibitor, AR overexpression and miR‐205 mimic +AR overexpression determined by Western blot analysis; F, the protein levels of miR‐205 and AR in cells treated with miR‐205 mimic, miR‐205 inhibitor, AR overexpression, and miR‐205 mimic +AR overexpression determined by Western blot analysis; *, *P* < 0.05 vs the NC mimic group or the Bio‐probe NC group or the control and NC groups; measurement data were expressed as mean ± SD; data in each group were analysed with ANOVA; the experiment was repeated three times; DMED, erectile dysfunction rats with diabetic mellitus; miR‐205, microRNA‐205; AR, androgen receptor; NC, negative control; 3'‐UTR, 3'‐untranslated region; qRT‐PCR, quantitative real‐time polymerase chain reaction.

According to analyses by the online target gene prediction software, microRNA.org, a specific binding area exists between the AR gene sequence and miR‐205, indicating that AR is a potential target gene of miR‐205 (Figure 5A). We further confirmed the relationship between miR‐205 and AR by using a dual‐luciferase reporter gene assay. In comparison with the NC group, the luciferase activity was found to be significantly decreased in Wt‐smiR‐205/AR co‐transfection of the miR‐205 mimic transfection group (*P* < 0.05), while there were no significant differences in terms of the luciferase activity of MUT 3’‐UTR (*P* > 0.05) (Figure 5B), suggesting that miR‐205 could specifically bind to the AR gene.

As shown in Figure 5C, the expression of AR in the Bio‐miR‐205‐WT (CSMCs transfected with WT biotinylated miR‐205) was demonstrated to be increased in comparison with the Bio‐probe NC group (*P* < 0.05), and that in the Bio‐miR‐205‐MUT (CSMCs transfected with MUT biotinylated miR‐205) presented with no significant differences (*P* > 0.05). This finding suggested that Bio‐miR‐205‐WT can attract the AR gene to surround itself, while Bio‐miR‐205‐MUT lost the ability. These results indicated that the AR gene was the target gene of miR‐205, and could specifically bind to the WT miR‐205.

Next, qRT‐PCR and Western blot analysis were employed in order to investigate the regulation of miR‐205 on AR There was no statistical significance in miR‐205 and AR expression (*P* > 0.05) between the control and NC group. In comparison with the control group and the NC group, the miR‐205 expression in the miR‐205 mimic group was found to be increased, in addition to decreased expressions of AR expression; the miR‐205 expression in the miR‐205 inhibitor group was found to be decreased while the AR expression was increased (all *P* < 0.05). In addition, it was found that the expression of miR‐205 was not affected by AR overexpression (*P* > 0.05). In the miR‐205 mimic +AR overexpression group, the expression of miR‐205 was found to be decreased (*P* < 0.05), and AR expression manifested no statistical significance (*P* > 0.05) (Figure 5D‐F). These results suggested that miR‐205 might down‐regulate the expression of AR

### 3.6 MiR‐205 down‐regulation or AR overexpression promotes proliferation and inhibits apoptosis of CSMCs in vitro

**Figure 6:**
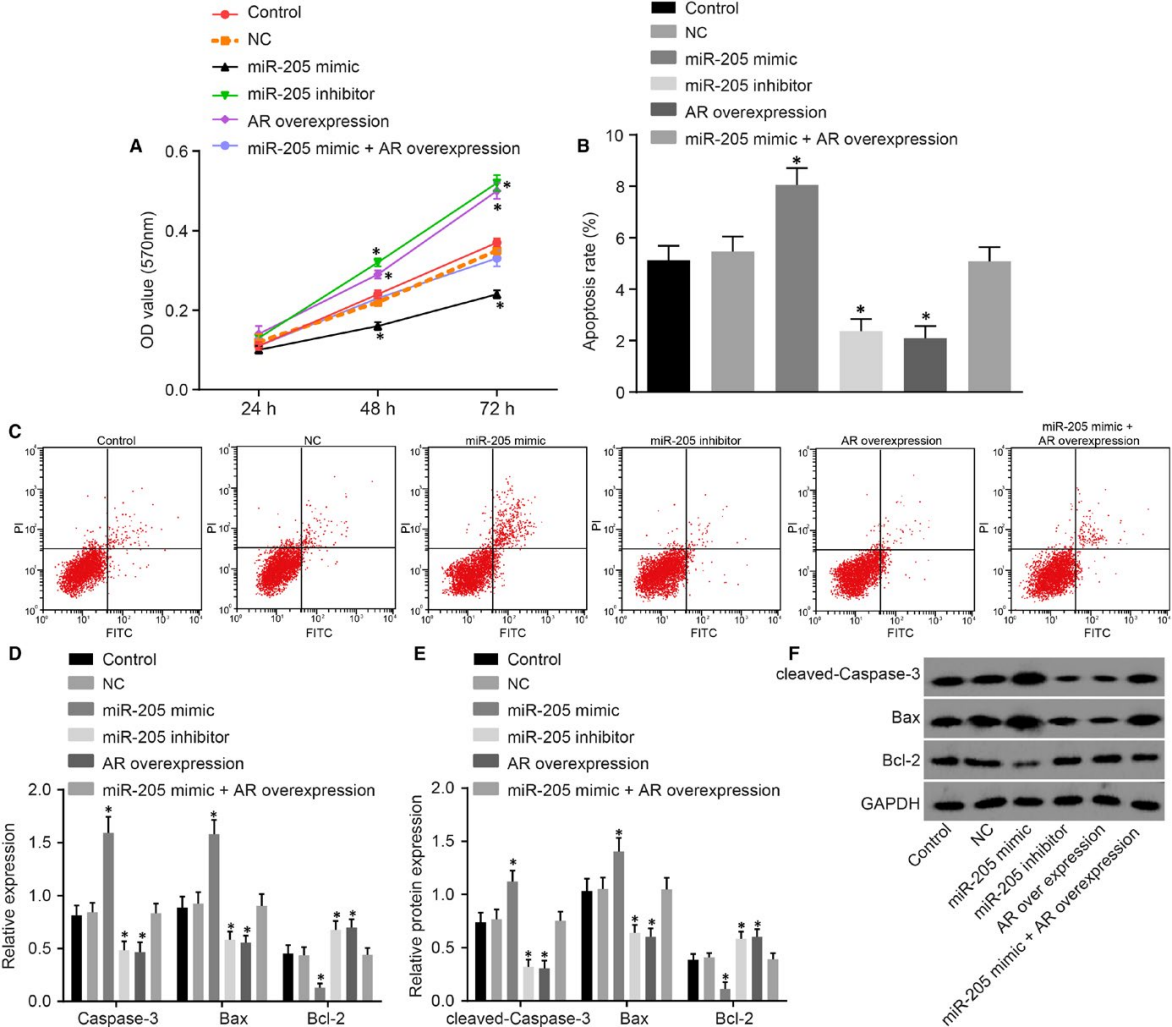
miR‐205 down‐regulation or AR overexpression enhances proliferation and suppresses apoptosis of CSMCs in vitro. A, cell viability detected by MTT assay; B, apoptosis rate analysis for CSMCs in response to the treatment of miR‐205 mimic, miR‐205 inhibitor, AR overexpression, and miR‐205 mimic +AR overexpression determined by flow cytometry; C, flow cytometry map demonstrating CSMC apoptosis condition; D, the mRNA levels of Caspase‐3, Bax and Bcl‐2 in CSMCs treated with miR‐205 mimic, miR‐205 inhibitor, AR overexpression, and miR‐205 mimic +AR overexpression determined by qRT‐PCR; E, the protein levels of Caspases3, Bax and Bcl‐2 in CSMCs treated with miR‐205 mimic, miR‐205 inhibitor, AR overexpression, and miR‐205 mimic +AR overexpression determined by Western blot analysis; F, the grey value of Caspases3, Bax and Bcl‐2 protein bands in CSMCs treated with miR‐205 mimic, miR‐205 inhibitor, AR overexpression, and miR‐205 mimic +AR overexpression determined by Western blot analysis; *, *P* < 0.05 *vs.* the control group and the NC group; measurement data were expressed as mean ± SD; data in each group were analysed with repeated measurement or ANOVA; the experiment was repeated three times; erectile dysfunction rats with diabetic mellitus; miR‐205, microRNA‐205; AR, androgen receptor; NC, negative control; CSMC, cavernous smooth muscle cells; MTT, 3‐(4, 5‐dimethyl‐2‐thiazolyl)‐2, 5‐diphenyl‐2‐H‐tetrazolium bromide; qRT‐PCR, quantitative real‐time polymerase chain reaction.

MTT assay was performed in order to investigate the role of miR‐205 or AR in the proliferation of CSMCs. Under the same initial cell concentrations, the OD value of the smooth muscle cells of the cavernous body in each group was found to be not statistically significant at the 24th hour time interval (*P* > 0.05). We found that cell proliferation among the control, NC and miR‐205 mimic +AR overexpression groups was of no statistical significance after 24, 48 or 72 hours of transfection (*P* > 0.05). In comparison with the control group and the NC group, at the 48th hours and 72nd hours time interval after transfection, the OD values of cells in the miR‐205 mimic group were found to be remarkably decreased, while the OD values in the miR‐205 inhibitor group and the AR overexpression group were increased (all *P* < 0.05) (Figure 6A).

Additionally, flow cytometry was employed in order to measure the apoptosis of CSMCs. There were insignificant differences in cell apoptosis among the control, NC and miR‐205 mimic +AR overexpression groups (*P* > 0.05). Compared with the control group and the NC group, the apoptosis rate of CSMCs in the miR‐205 mimic group was found to be significantly increased, while decreased rates were observed in the miR‐205 inhibitor group and the AR overexpression group (all *P* < 0.05) (Figure 6B,C).

Moreover, the effect of miR‐205 or AR on expression of Bax and Bcl‐2 in CSMCs in vitro was detected. The difference of the expression among the control, NC and miR‐205 mimic +AR overexpression groups was of no statistical significance (*P* > 0.05). In comparison with the control group and the NC group, the expression of Caspase‐3 and Bax in the miR‐205 mimic group was found to be significantly increased, while the expression of Bcl‐2 was significantly decreased. The expressions of Caspase‐3 and Bax were found to be significantly decreased in the miR‐205 inhibitor group and the AR overexpression group, in addition to increased Bcl‐2 expressions (all *P* < 0.05) (Figure 6D‐F). The aforementioned results suggested that down‐regulation of miR‐205 and up‐regulation of AR expression could promote proliferation as well as inhibit apoptosis of CSMCs.

## 4 DISCUSSION

Erectile dysfunction is a widespread condition affecting diabetic males, with prevalence rates as high as 85%.^23^ DM is considered to be a major risk factor for the onset of ED; however, the underlying pathogenesis of DMED remains to be largely unknown. Interestingly, miRs, which are novel and potent mediators of tissue injury, and their correlations with various benign urologic pathologies, specifically ED, remain unexplored.^24^ Therefore, owing to various evidence and findings, the current study aimed to explore the role of miR‐205 in DMED using multiple modes of testing and experimentation. In the current study, we demonstrated that miR‐205 contributed to the pathogenesis of DMED by negatively regulating AR Additionally, it was revealed that AR is a target gene of miR‐205, and down‐regulation of miR‐205 or up‐regulation of AR significantly improved the erectile functioning of rats suffering from DMED by promoting proliferation as well as inhibiting apoptosis of CSMCs.

Initially, the current study revealed that compared with normal rats, rats with DMED exhibited significantly down‐regulated expressions of AR, and significantly up‐regulated miR‐205 expressions. Interestingly, low androgen and AR levels in elderly men have been reported to be involved in the incidence and development of DM through interaction with obesity, glucose homeostasis as well as insulin resistance.^17^ Next, this study found that miR‐205 up‐regulation was responsible for a decrease in AR expressions in rats with DMED. Subsequently, AR was identified to be a target gene of miR‐205. This particular finding was in accordance with a previous study on PC, in which the expression of miR‐205 was found to be negatively correlated with the expression of AR.^17^ The most significant effect of miR‐205 on cell growth in prostate cells is dependent on androgen.^25^

Essentially, the findings of the current study indicate that down‐regulation of miR‐205 or up‐regulation of AR can alleviate the erectile function of rats with DMED. According to previous evidence, decreed expressions of AR were strongly associated with ED, leading to inadequate penile arterial blood flow, one of the primary causes of ED.^26^ Low androgen levels in the neonatal period changed gene expression of smooth muscle cell differentiation‐related biomarkers, which resulted in a decline of CSMCs, and consequently to penile dysfunction.^27^ In addition, endothelial progenitor cells (EPC) markers were expressed in the cavernosal sinusoidal endothelial space.^28^ It is known that androgens stimulate EPC through an AR‐mediated pathway.^29^ Several studies have shown that patients suffering from ED exhibit a decreasing number of circulating EPCs.^30^, ^31^ These results suggest that high expression of AR may improve the erectile function.^28^ Suppression of miR‐205 alone was correlated selectively with increased 3 T3‐L1 cell proliferation.^32^ In a previous study, several miRNAs were confirmed to be aberrantly expressed in the corpus cavernosum of aging rats with ED, including miR‐1, miR‐200a, miR‐203 and miR‐206.^33^ These aforementioned miRNAs play potent roles via regulation of endothelial nitric oxide (NO) synthase, NO, protein kinase G, the prostaglandin E1 and protein kinase A pathways, and thus lead to the development of aging‐induced ED in rats.^33^, ^34^

Another finding of the current study is that down‐regulated expression of miR‐205 or up‐regulated expression of AR can prevent tissue fibrosis in the corpus cavernosum. Furthermore, it was also unfounded that overexpression of AR could rescue miR‐205 up‐regulation mediated DMED. In addition, emerging data suggest that miRNAs participate in the regulation of fibrosis process.^35^ Several recent studies have identified that the miR‐200 family, including miR‐200a, miR‐200b, miR‐200c, miR‐141, miR‐149 and miR‐205, function as key regulators of epithelial‐mesenchymal transition, from which the myofibroblast, the key cellular mediator of fibrosis, is derived.^36^, ^37^ Moreover, lipoxin A4 was also thought to be effective in inhibiting corporal fibrosis, thus improving ED in rats with type I diabetes.^38^ Androgen deficiency induces corporal fibrosis through the activation of the Smad and non‐Smad pathways, characterized by loss of corpus cavernosum smooth muscles cells, corresponding reduction in SM/C ratio, and accumulation of ECM proteins.^39^ Moreover, it was also further reported that androgen deprivation leads to penile tissue atrophy, alterations in dorsal nerve structure, alterations in endothelial morphology, reduction in trabecular smooth muscle cells content, increase in deposition of extracellular matrix and accumulation of fat‐containing cells (adipocytes) in the subtunical region of corpus cavernosum.^40^

In summary, findings of the current study lead to the conclusion that erectile functioning in rats suffering from DM can be improved by down‐regulating the expression of miR‐205 or up‐regulating the expression of AR in DMED. However, further research is warranted in order to explore the mechanism of AR participation in the downstream pathways of ED.

## CONFLICT OF INTEREST

The authors have declared no conflicts of interests with respect to the research, authorship and/or publication of this article.
