# MicroRNA‐205 is associated with diabetes mellitus‐induced erectile dysfunction via down‐regulating the androgen receptor

**DOI:** 10.1111/jcmm.17720

**Published:** 2023-03-20

**Correction:** Because of mistakes in the selection of pictures when I set up them, partial overlap was in the two pictures. I would like to hope you to replace this picture.
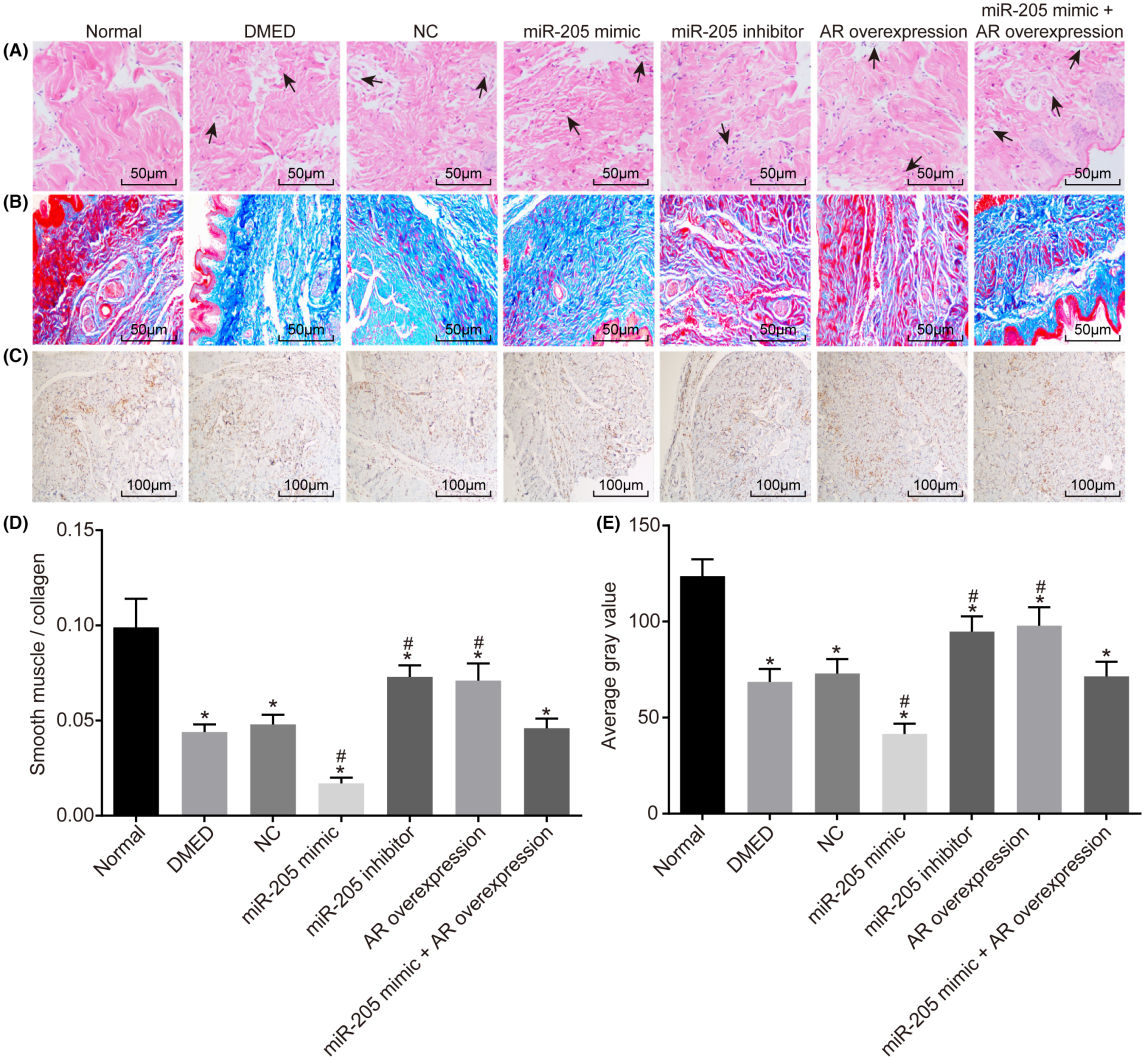
FIGURE 3
