# Supplementary figures and images for: MicroRNA‐205 is associated with diabetes mellitus‐induced erectile dysfunction via down‐regulating the androgen receptor
Source: J Cell Mol Med. 2019 Feb 7;23(5):3257–70. doi: 10.1111/jcmm.14212 (PMC6484320; doi:10.1111/jcmm.14212)

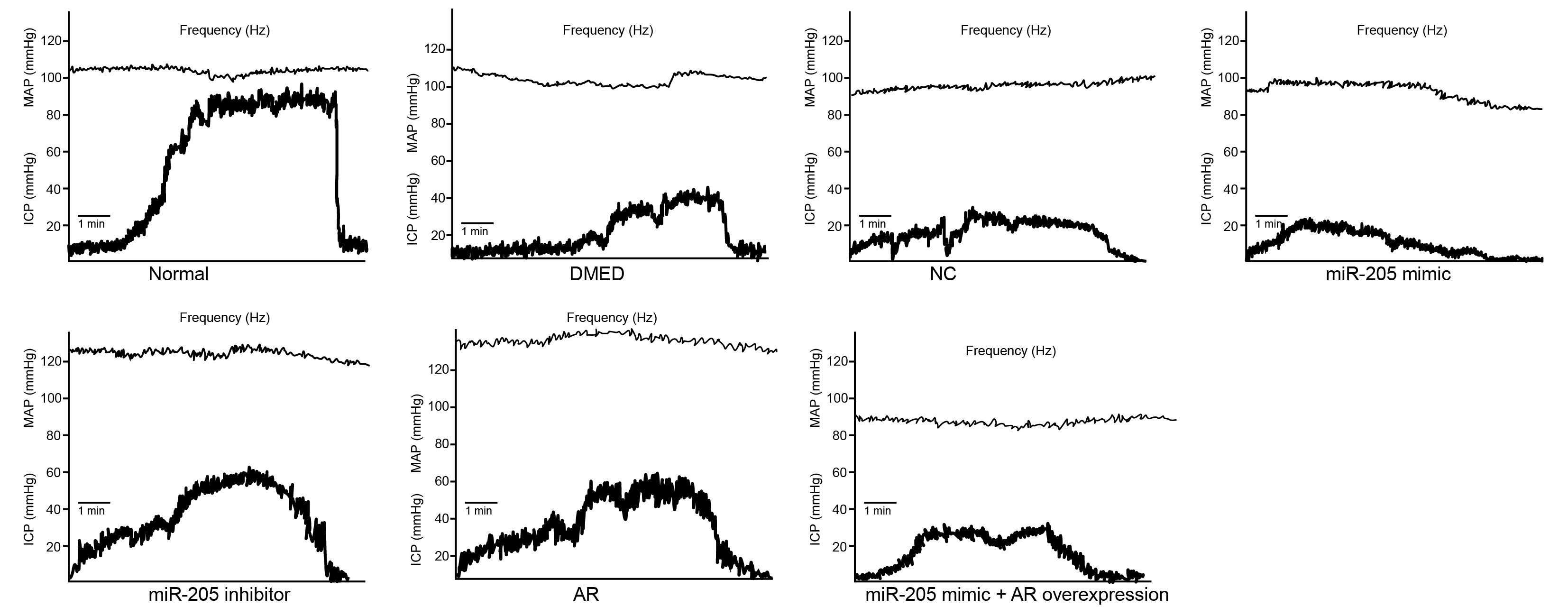

Supplement: Supplementary file 1 [file JCMM-23-3257-s001.jpg]

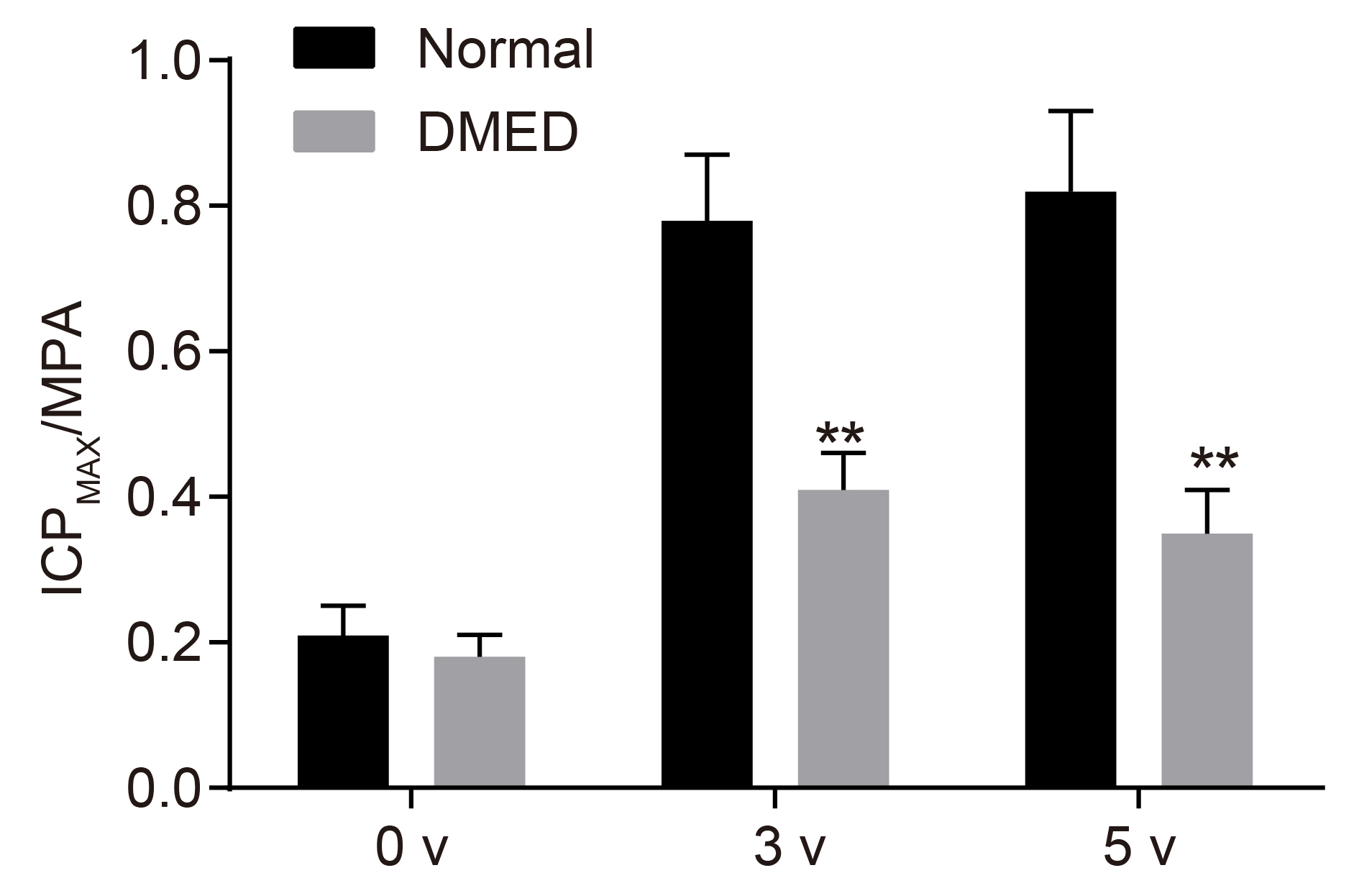

Supplement: Supplementary file 2 [file JCMM-23-3257-s002.jpg]
