# Supplementary material for: MicroRNA‐205 is associated with diabetes mellitus‐induced erectile dysfunction via down‐regulating the androgen receptor
Source: J Cell Mol Med. 2019 Feb 7;23(5):3257–70. doi: 10.1111/jcmm.14212 (PMC6484320; doi:10.1111/jcmm.14212)
Supplement: Supplementary file 3 [file JCMM-23-3257-s003.doc]

**SUPPLEMENTARY TABLE** **1** Serum testosterone concentration is lowered by miR-205downregulation in rats with DMED

| Group | Number | Testosterone concentration (nmol/L) | |
| --- | --- | --- | --- |
|  |  | Before STZ induction | After STZ induction |
| Normal | 10 | 177.62 ± 55.23 | 197.03 ± 60.25 |
| DMED | 10 | 171.09 ± 56.34 | 94.15 ± 28.98* |
| NC | 10 | 170.23 ± 52.08 | 92.04 ± 27.05* |
| miR-205 mimic | 10 | 165.34 ± 50.12 | 42.18 ± 15.08*# |
| miR-205 inhibitor | 10 | 178.38 ± 58.25 | 148.87 ± 34.97*# |
| AR | 10 | 179.54 ± 59.05 | 146.26 ± 35.45*# |
| miR-205 mimic + AR Over-expression | 10 | 180.38 ± 57.49 | 96.54 ± 27.47* |

Notes: *, *p* < 0.05 *vs.* the normal group; #, *p* < 0.05 *vs.* the DMED group and the NC group; STZ, streptozocin; DMED, erectile dysfunction rats with diabetic mellitus; miR-205, microRNA-205; AR, androgen receptor.
